# When the Next Disease Strikes: How To Communicate (and How Not To)

**DOI:** 10.1089/hs.2016.0100

**Published:** 2017-02-01

**Authors:** Tara Kirk Sell

In an increasingly interconnected world, the potential for infectious diseases to spread internationally is an inescapable fact. The country will face one or more infectious disease health threats over the course of the next administration. Dealing with these threats will require science-based assessment, judicious management, and effective risk communication.

Over the past 8 years, a number of health threats have affected the United States, and public officials have struggled to communicate effectively. During the 2014 Ebola outbreak, some depictions of the threat Ebola posed to the United States caused the public to become much more concerned about the disease than was called for (only 2 people were infected on US soil and both were healthcare workers in direct contact with a patient). During the 2016 Zika threat, we've seen that people have a mixed understanding of the disease and the protective actions they should take, despite months of efforts to inform them. The incoming Administration and Congress will be called on to communicate with the public during similar periods of crisis, potentially ranging from naturally occurring disease outbreaks to intentional bioterrorism, in the future.

## Why Is Risk Communication Important?

No matter how effective the planned response, poor communication with the public will greatly undermine its effectiveness. In fact, poor communication could exacerbate the threat and lead to greater disease effects as well as loss of trust in the government's ability to protect the nation in the future. Even a small crisis can spiral out of control without appropriate risk communication, and effective risk communication during a significant event could have a large life-or-death impact.

Failure to communicate well may lead to the perception that the government is unable to respond to the outbreak. For instance, poor communication during Hurricane Katrina led to avoidable deaths, exacerbation of existing racial tensions, and loss of faith in government at all levels. In contrast, using communication practices that have been proven to be effective sets the stage for effective crisis management and helps the public act in ways that help themselves and their neighbors emerge from a crisis. For example, during the H1N1 outbreak, a trusted and reliable spokesperson communicated well about what was known, what was unknown, and what people should do to protect themselves. These messages were well received.

## Key Principles in Effective Communication

Public health agencies have increasingly embraced risk communication as an important aspect of emergency response and have expanded efforts to improve the effectiveness of their efforts. For instance, the Centers for Disease Control and Prevention (CDC) have adopted Crisis and Emergency Risk Communication (CERC) practices as central to their response, which stress that successful communication is the first source of information, is accurate, is credible, shows empathy, promotes action, and shows respect.^1^

There are many different descriptions of basic risk communication principles, but they all have a number of concepts in common, including the ability to adapt to a changing situation, understanding the audience, and building trust. Risk communication should be done with the goal of helping people become informed about a potential risk so that they can make appropriate decisions to protect their health, understand response activities, and potentially assist in community efforts to address the risk. Good risk communication practices take into account individual and community knowledge, attitudes, and risk perception and tailor messages to ensure that people are informed about and given concrete steps to protect themselves from infectious diseases.

## Aspects of Risk Perception

Regardless of measured and quantitative or technical risk assessment, several factors can make health threats seem more “risky” to the public. Risks that are unknown—meaning they are not observable, unknown to those exposed, delayed in effect, new, or unknown to science—and risks that are highly dreaded—meaning they are uncontrollable, catastrophic, fatal, not equitable, high risk to future generations, not easily reduced, increasing in risk, or involuntary—are perceived to be more severe.^2^ For instance, people often perceive nuclear energy to be very high risk, but they do not assign similarly high risks to the consumption of alcoholic beverages, even though actual risks from nuclear energy are comparatively very low. However, people are much more familiar with alcoholic beverages, which have a more immediate effect, are controllable, and are voluntarily ingested. In contrast, nuclear power could potentially present risks from radiation that are delayed, unobservable, uncontrollable, involuntary, catastrophic, fatal, and dangerous to future generations. As a result, the latter is perceived to be higher risk than the former. In essence, public outrage plays an important role in risk perception.^3^

When communicating about health threats, officials should be aware of these factors and understand how they will influence the public's reaction. For example, although the intense public reaction to Ebola in the United States was not warranted, considering the specific conditions required for spread of the disease, the fatal and dreaded nature of the disease increased perception of risk. In the case of Zika, the disease can have severe impacts on unborn fetuses, which increases public perception of risk from the disease. Heightened perceptions of risk, regardless of their basis in real risks to the public, need to be accepted as valid concerns to be addressed.

## Recommendations

➢ **Don't be taken by surprise.**

Expect to respond to and communicate about infectious disease threats during this coming Administration. The American public will expect the government to be prepared to have an effective response when such an event occurs, whether it is a bioterrorist attack or a naturally occurring pandemic, and to communicate rapidly and transparently about it. This means that the government will need to provide timely information about what is being done and what is known and not known. A number of widely accepted steps are currently being done, such as maintaining situational awareness about infectious diseases that may spread to the United States, preparing easily modifiable message templates for both intentionally caused and naturally occurring events, and identifying and training spokespeople who are comfortable relaying complex information effectively. Furthermore, establishing an advisory panel ahead of time of people with in-depth understanding of infectious disease and public health practices may help policymakers determine the most appropriate public health response in an emergency.

➢ **Communicate uncertainty when it exists.**

It is impossible to know exactly how an infectious disease health crisis will unfold, especially in the event of a new or deliberate release of an infectious agent. Communication that is overly certain and then is perceived as “wrong” can significantly undermine public trust and the likelihood that future messages will be accepted and acted upon. Past experience has shown that being clear about what is unknown, what work is still being done to increase understanding, and that information may change helps increase acceptance of messages.

➢ **Take a deep breath: Carefully consider and communicate policy responses.**

During the next infectious disease health threat, many policymakers, the public, and the media will expect rapid response. However, first impulses for response often require more measured consideration to ensure that they will actually protect health. For instance, it is best to use travel bans, quarantines, and school closures sparingly. Although these sound like effective measures, second and third order effects often undermine them to the point of causing more harm than good. Communicating these complicated relationships will be important to response efforts. Transparent communication about what is being done to determine the best path forward can help to relieve some of the pressure that may lead to choosing a suboptimal response.

➢ **Eliminate the phrase “out of an abundance of caution.”**

Use of this phrase can be a prime indicator that a certain activity isn't warranted by science. What is being communicated is that a certain action may not be necessary but is going to be done anyway just to be overly careful. However, this assumes that there are no consequences for overly cautious and unnecessary actions. In fact, there are consequences to some public health actions that have been taken in epidemic crises—beyond the economic costs, there can be restrictions on civil liberties and loss of resources. In addition, use of this phrase and the activities that go along with it can undermine messages about risks being based in science and fact, and they could suggest to the public that they shouldn't trust science and official recommendations. Instead, a transparent approach that remains consistent and honest about potential risks is needed.

➢ **Do more research on what works.**

CDC and a number of other federal agencies do high-quality research on existing communication practices and have greatly improved risk communication by the federal government. This work has improved the research, practice, and training base for risk communication, but gaps remain. Improvements in risk communication need to be maintained through continual preparation and research; otherwise, progress and expertise will be lost. Furthermore, continued efforts to improve knowledge and practice surrounding what makes up good risk communication are needed.

Continued efforts to develop communication research and products at the federal agency level are important to enabling an effective response to the next infectious disease threat. This not only improves federal communication during infectious disease emergencies, but also results in guidance and best practices that are shared with state and local agencies to improve response at the source.

## Conclusion

Effective risk communication is essential in response to infectious disease health threats. These types of threats will affect the next Administration, which will need to have made a number of key investments to respond effectively. Among these investments are appropriate funding for communication efforts at CDC and others at the federal agency level and in state and local governments. In addition, the next Administration should embrace a number of communication practices, including being explicit about uncertainty and avoiding the use of the phrase “out of an abundance of caution.”

Furthermore, public health response activities should be carefully considered and transparently communicated with end goals in mind. The stakes of effective risk communication are high. Successful communication and response during the next infectious disease health threat will be important in ensuring public faith and trust that the next Administration can effectively manage threats to the country. Getting risk communication right during the next disease threat will protect the lives of Americans and help to establish the ability of the next Administration to lead effectively during crisis.
